# Associations of non-traditional lipid parameters with high-risk plaques characterized by optical coherence tomography in acute myocardial infarction culprit lesions

**DOI:** 10.3389/fcvm.2026.1698482

**Published:** 2026-01-29

**Authors:** Mengyao Cheng, Erkun Xing, Minmin Wang, Lixia Zhang, Zheng Zhang

**Affiliations:** 1 The First Clinical Medical College, Lanzhou University, Lanzhou, Gansu, China; 2 Heart Center, The First Hospital of Lanzhou University, Lanzhou, Gansu, China

**Keywords:** acute myocardial infarction, apolipoprotein, cholesterol, coronary plaque, optical coherence tomography

## Abstract

**Objective:**

The objective of this research was to investigate the association between non-traditional lipid parameters and optical coherence tomography (OCT)-characterized high-risk plaques in patients with acute myocardial infarction (AMI).

**Methods:**

This retrospective study included 249 first-episode AMI patients admitted to the First Affiliated Hospital of Lanzhou University between January 2022 and December 2024. All patients underwent OCT-guided assessment of culprit lesions before revascularization. High-risk plaques were defined by more than two of the following features: lipid arc ≥90 °, fibrous cap thickness <65 μm, or plaque rupture/thrombus. Lesions with fewer than two of these criteria were classified as non-high-risk plaques. Clinical and laboratory data were collected, and a comprehensive lipid profile was calculated, including traditional indicators [e.g., non-HDL cholesterol (non-HDL-C)] and non-traditional ratios [e.g., apolipoprotein B/A1 ratio (ApoB/A1)]. Spearman correlation was used to assess relationships between lipid parameters and high-risk plaques. After excluding collinear variables, logistic regression, restricted cubic spline (RCS), and subgroup analyses were performed. Model discrimination and clinical value were evaluated using receiver operating characteristic (ROC) curves, the DeLong test, integrated discrimination improvement (IDI), net reclassification index (NRI), and decision curve analysis (DCA).

**Results:**

Among 249 AMI patients, 137 (55.0%) exhibited OCT-characterized high-risk plaques. These patients were more often male (89.8%) and presented with STEMI (84.7%). They had elevated levels of myoglobin, LDL-C, non-HDL-C, ApoB, ApoB/A1, remnant lipoprotein cholesterol (RLP-C), non-HDL-C/HDL-C ratio (NHHR), and TC/HDL-C (all *P* < 0.05). OCT features included thinner fibrous caps, smaller lumen areas, larger lipid arcs, and higher incidences of rupture, erosion, thrombus, macrophage infiltration, cholesterol crystals, and calcification (all *P* < 0.05). Both ApoB/A1 (OR = 3.688, 95% CI: 1.211–11.230) and non-HDL-C (OR = 3.023, 95% CI: 1.238–7.378) were independently and linearly associated with high-risk plaques. No significant interactions were observed across clinical subgroups (all *P* for interaction > 0.05). The combined model incorporating the two markers achieved the highest discriminative performance (AUC = 0.696) and significantly improved the baseline model (DeLong test *P* < 0.05), with additional gains confirmed by IDI, NRI, and DCA (all *P* < 0.05).

**Conclusion:**

Both the non-traditional ApoB/A1 ratio and the traditional lipid marker non-HDL-C were independently and linearly associated with OCT-characterized high-risk plaques in AMI. Their combined assessment enhanced the identification of high-risk plaques morphology.

## Introduction

Acute myocardial infarction (AMI) remains a major global cause of cardiovascular mortality, primarily driven by the rupture or erosion of vulnerable coronary plaques (1). Lipid metabolic dysregulation plays a central role in plaque formation and destabilization, influencing both inflammatory activity and thrombogenicity (2). Although low-density lipoprotein cholesterol (LDL-C) is the cornerstone target in atherosclerotic management (3), its value in identifying high-risk plaque phenotypes is limited. Acute-phase LDL-C levels fluctuate under inflammatory stress—such as IL-6-mediated suppression of hepatic LDL receptor expression—reducing their reliability for risk assessment (4). Moreover, traditional LDL-C measurements fail to reflect the compositional and functional diversity of atherogenic lipoproteins, including apolipoprotein composition and oxidative modifications, which critically determine plaque vulnerability (5).

In recent years, non-traditional lipid parameters have shown superior potential in capturing residual atherogenic risk and reflecting the lipid–inflammation–plaque interaction. The apolipoprotein B/A1 ratio (ApoB/A1) represents the balance between pro-atherogenic ApoB-containing lipoproteins and protective ApoA1-rich HDL, demonstrating stronger associations with cardiovascular events than conventional lipids (6). Similarly, the non-HDL-C/HDL-C ratio (NHHR) and total cholesterol/HDL-C ratio (TC/HDL) integrate the total burden of atherogenic particles and have been linked to plaque instability (7–9). Notably, the constituent marker non-HDL-C represents a conventional lipid parameter that quantifies the cholesterol content of all ApoB-containing atherogenic lipoproteins, whereas its ratio-based derivatives (e.g., NHHR and TC/HDL) are considered non-traditional parameters that extend beyond standard lipid measurements. In addition, markers such as remnant lipoprotein cholesterol (RLP-C) and the lipoprotein combine index (LCI) further bridge lipid metabolic pathways with endothelial dysfunction and inflammatory activation (10, 11).

However, evidence directly linking these lipid markers to OCT-characterized high-risk plaque morphology remains unclear. Therefore, this study investigated the associations between non-traditional lipid parameters and OCT-characterized high-risk plaques in AMI culprit lesions, aiming to elucidate their potential value for lipid-based risk stratification and individualized management.

## Methods

### Patient population

**Figure 1:**
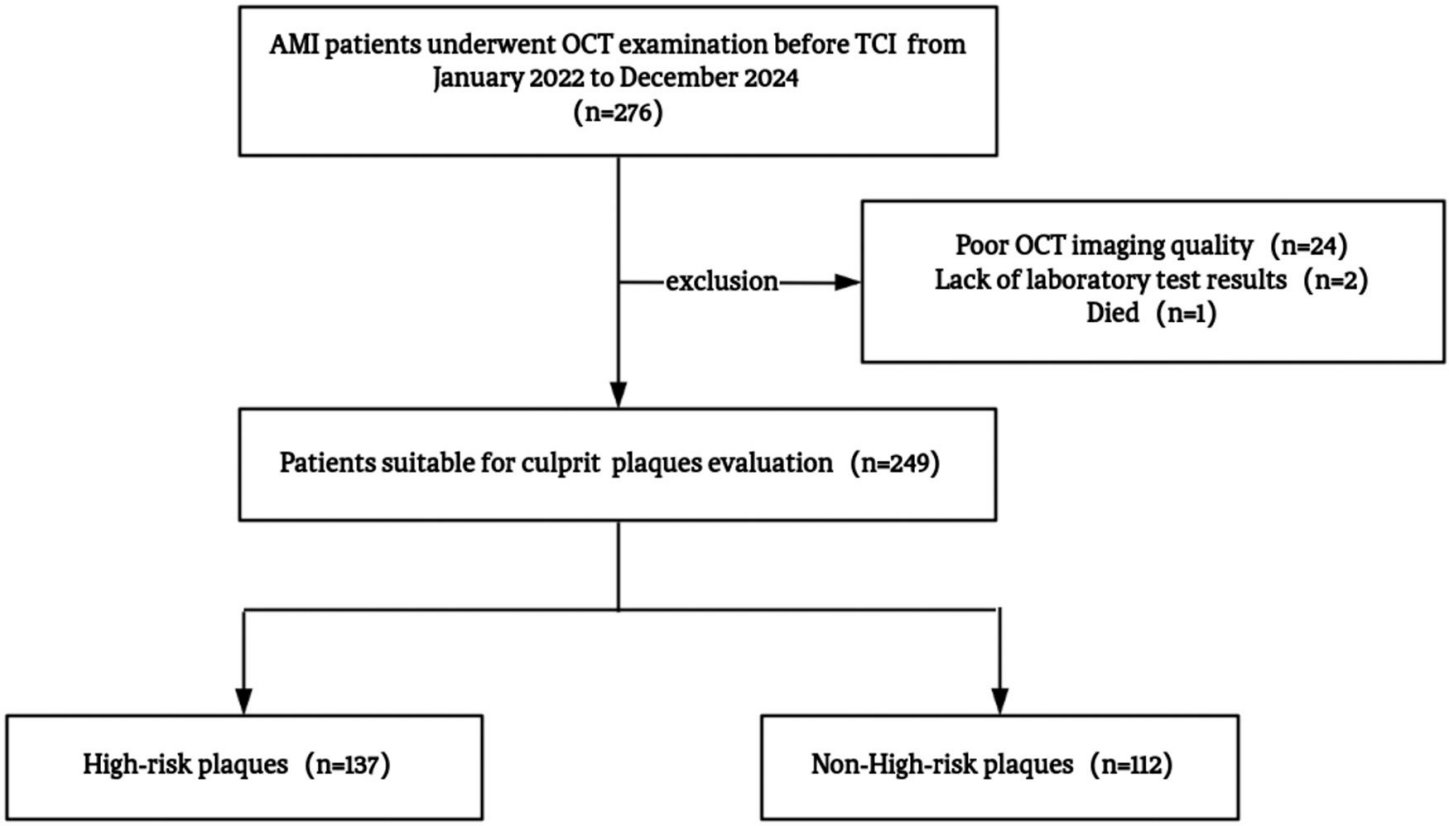
Study flowchart. AMI, acute myocardial infarction; OCT, optical coherence tomography; TCI, transcatheter coronary intervention.

This retrospective cross-sectional study enrolled first-episode AMI patients admitted to the First Affiliated Hospital of Lanzhou University between January 2022 and December 2024. AMI was diagnosed according to the Fourth Universal Definition (12), requiring cardiac troponin I elevation above the 99th percentile with dynamic changes and objective evidence of myocardial ischemia, including typical chest pain ≥20 min, new ECG changes, wall motion abnormalities, or angiographic thrombotic occlusion. All patients underwent optical coherence tomography (OCT) imaging of culprit lesions before revascularization. Exclusion criteria included (1) recurrent AMI within 30 days, contrast allergy, or type II, IV, or V AMI; (2) cardiogenic shock (Killip IV), severe renal dysfunction (eGFR <30 mL/min/1.73 m^2^), hepatic decompensation, active malignancy, or systemic inflammation; (3) left main disease, chronic total occlusion, severe calcification (calcium arc > 270°), or marked vessel tortuosity (>90°); and (4) poor OCT image quality (<80% lesion coverage or >50% attenuation). After excluding 24 cases with poor OCT quality, two with missing data, and one in-hospital death, a total of 249 patients were included in the final analysis (Figure 1).

### Coronary angiography and OCT imaging analysis

Diagnostic coronary angiography was performed via femoral or radial access using 6F or 7F catheters. Each major coronary vessel was imaged in at least two orthogonal projections to identify the infarct-related (culprit) artery according to clinical presentation, ECG findings, and angiographic evidence. In patients with STEMI, limited manual thrombus aspiration or gentle balloon pre-dilation was occasionally performed to ensure adequate lumen visualization. All interventions were conducted with low pressure and over short durations to minimize plaque disruption, which has been shown to exert negligible influence on plaque morphology according to the IWG-OCT consensus (13–15).

OCT imaging was performed using a frequency-domain system (C7-XR, Abbott Vascular, Santa Clara, CA, USA; axial resolution 12–15 μm, lateral 20–25 μm) following standardized intracoronary protocols. After administration of intracoronary nitroglycerin (200 μg), culprit lesions were imaged using automated pullback at 2.5 mm/s under continuous flushing (contrast:saline = 7:3, 3.0 mL/s). All images were analyzed offline by two independent, blinded cardiologists (Offline Review Workstation v5.1, Abbott Vascular), and discrepancies were adjudicated by a senior expert following the International Working Group for Intravascular OCT Standardization (IWG-OCT) recommendations (14, 16). The culprit plaque was defined as the segment centered on the culprit lesion and extending ≥5 mm proximally and distally to the nearest normal vessel segment. Based on established OCT criteria, plaque rupture (PR) was defined as fibrous cap discontinuity with an underlying cavity, whereas plaque erosion (PE) was characterized by the presence of thrombus attached to an irregular luminal surface without evidence of cap rupture in multiple consecutive frames. Thrombus was identified as an irregular mass protruding into or adjacent to the lumen. For fibroatheroma, fibrous cap thickness was measured three times at the thinnest site and averaged. Thin-cap fibroatheroma (TCFA) was defined as a fibroatheroma with cap thickness <65 μm. Lipid length was determined as the longitudinal extent of contiguous fibroatheroma cross-sections, and macrophage accumulation was identified as signal-rich punctate or confluent regions with higher intensity than background speckle noise and frame-to-frame variability.

**Figure 2:**
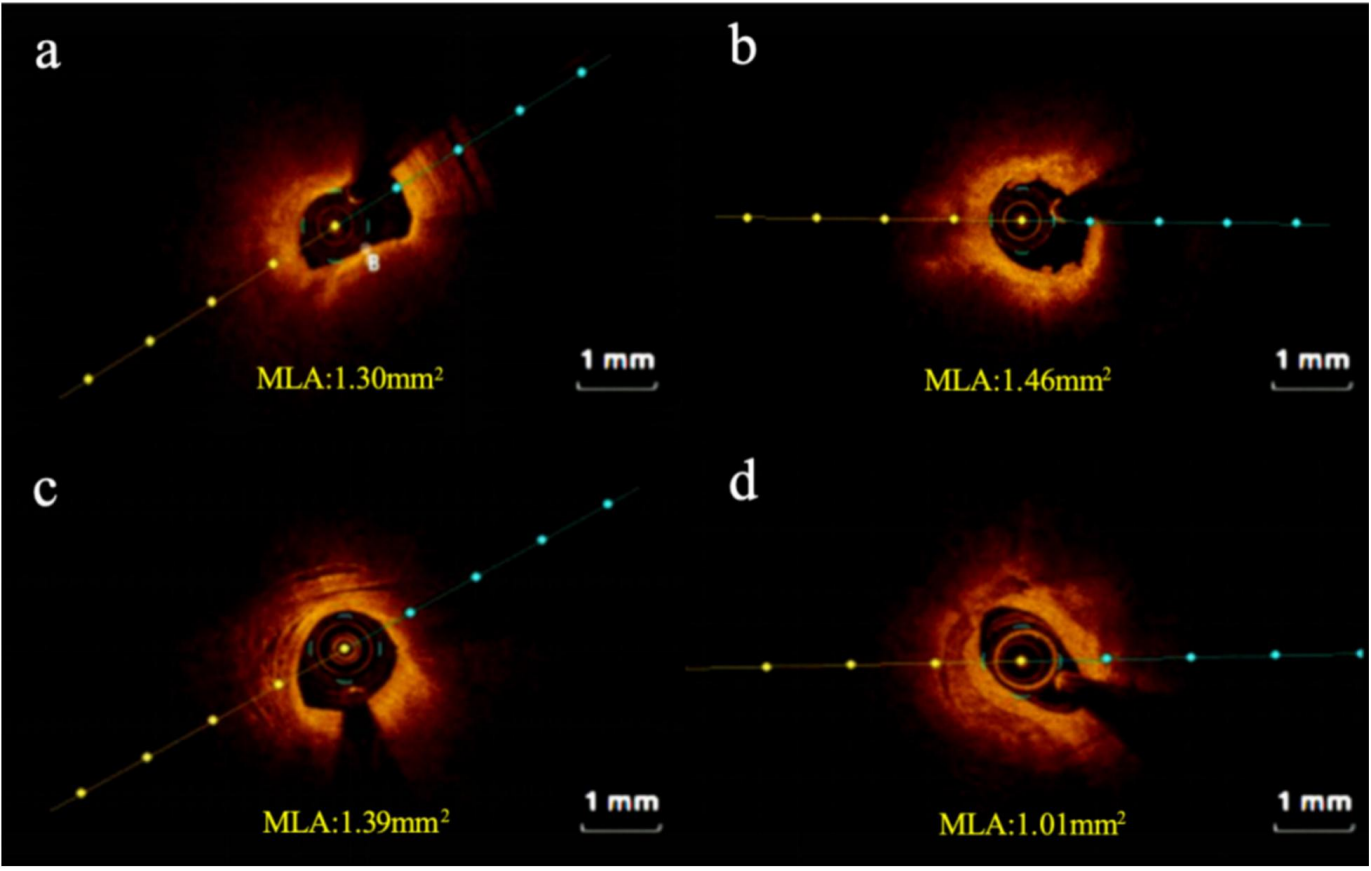
Representative cross-sectional optical coherence tomography images. **(a)** High-risk plaque in STEMI patient; **(b)** high-risk plaque in NSTEMI patient; **(c)** non-high-risk plaque in STEMI patient; **(d)** non-high-risk plaque in NSTEMI patient. STEMI, ST-segment elevation myocardial infarction; NSTEMI, Non-ST-segment elevation myocardial infarction; MLA: minimum lumen area.

High-risk plaques were defined as lesions fulfilling ≥2 of the following criteria: lipid arc ≥ 90°, fibrous cap thickness <65 μm, or the presence of plaque rupture/thrombus. Lesions with fewer than two features were classified as non-high-risk plaques. Representative OCT images of both categories are shown in Figure 2.

### Data collection

Demographic, clinical, and laboratory parameters were systematically extracted from the institutional electronic health record system using a standardized case report form. Baseline characteristics encompassed age at presentation, biological sex, body mass index (BMI), and documented cardiovascular risk factors, including hypertension, diabetes, smoking, and alcohol status. Information regarding prior use of lipid-lowering medications was also collected. Venous blood samples were collected under standardized phlebotomy protocols within 6 h of admission, before lipid-modifying therapy initiation. Comprehensive lipid data were collected, including a conventional lipid panel [total cholesterol (TC), triglycerides (TG), LDL-C, HDL-C, and non-HDL-C] and non-traditional lipid biomarkers [apolipoprotein B/A1 ratio (ApoB/A1), NHHR, total cholesterol/HDL cholesterol ratio (TC/HDL), HDL cholesterol to apolipoprotein A1 ratio (HDL-C/ApoA1), RLP-C (calculated as TC−HDL-C−LDL-C), and LCI (calculated as (TC × TG × LDL-C)/HDL-C)].

### Statistical analysis

All statistical analyses were performed using R version 4.3.1. Continuous variables were tested for normality using the Shapiro–Wilk test. Variables with a normal distribution were presented as mean ± standard deviation (SD) and compared between groups using the independent samples t-test. Non-normally distributed data were presented as median (interquartile range) and compared between groups using the Mann–Whitney U test, while categorical variables were expressed as counts (percentages) and compared using the *χ*^2^ test or Fisher's exact test, as appropriate. Correlations between lipid parameters and high-risk plaques were first evaluated by Spearman analysis. Variables with severe multicollinearity were excluded. Bidirectional logistic regression analyses were performed after collinearity diagnostics to identify factors associated with high-risk plaques, and robustness was verified through adjusted models. Restricted cubic spline (RCS) models were used to illustrate the dose–response relationships between lipid parameters and plaque risk. Subgroup analyses were performed according to age, BMI, sex, hypertension, diabetes, smoking status, AMI type (STEMI/NSTEMI), and plaque phenotype (PR/PE). Model discrimination was assessed using receiver operating characteristic (ROC) curves and compared by the DeLong test. Incremental predictive value was evaluated by the integrated discrimination improvement (IDI) and continuous net reclassification index (NRI). Decision curve analysis (DCA) was applied to assess clinical net benefit across varying threshold probabilities. A two-sided *P* < 0.05 was considered statistically significant.

## Results

### Baseline characteristics of the study population

A total of 249 patients with first-episode AMI were included in the final analysis, comprising 137 patients in the high-risk plaque group and 112 in the non-high-risk group. Compared with the non-high-risk group, the high-risk plaque group had a significantly higher proportion of male patients (89.78% vs. 80.36%, *P* = 0.035) and a greater prevalence of STEMI (84.67% vs. 68.75%, *P* = 0.003). Laboratory analyses revealed significantly elevated levels of myoglobin (*P* = 0.026) and lactate dehydrogenase (*P* < 0.001) in the high-risk group. Regarding lipid profiles, levels of LDL-C, non-HDL-C, ApoB, ApoB/ApoA1, RLP-C, NHHR, and TC/HDL were all markedly higher in the high-risk group (all *P* < 0.05) (Table 1).

**Table 1:** Baseline characteristics in non-high-risk and high-risk plaque groups.

Variables	All *N* = 249	Non-high-risk *N* = 112	High-risk *N* = 137	Statistic	*P-*value
Demographics					
Male, *n* (%)	213 (85.54)	90 (80.36)	123 (89.78)	*χ*^2^ = 4.425	0.035
Age, years	56.00 (49.00, 65.00)	59.00 (50.75, 65.25)	54.00 (48.00, 64.00)	Z = −1.670	0.095
BMI, kg/m^2^	24.97 (23.12, 26.95)	24.72 (23.28, 26.44)	25.30 (23.03, 27.04)	Z = −0.977	0.328
SBP (mmHg)	126.00 (111.00, 139.00)	127.00 (113.50, 137.00)	124.00 (109.00, 141.00)	Z = −0.732	0.464
DBP (mmHg)	80.00 (70.00, 90.00)	80.00 (70.00, 89.00)	80.00 (69.00, 92.00)	Z = - 0.245	0.806
Smoke, *n* (%)	141 (56.63)	57 (50.89)	84 (61.31)	χ^2^ = 2.725	0.099
Alcohol, *n* (%)	91 (36.55)	42 (37.50)	49 (35.77)	χ^2^ = 0.080	0.777
STEMI, *n* (%)	193 (77.51)	77 (68.75)	116 (84.67)	χ^2^ = 8.961	0.003
Killip, *n* (%)				-	0.209
1	193 (77.51)	90 (80.36)	103 (75.18)		
2	45 (18.07)	16 (14.29)	29 (21.17)		
3	6 (2.41)	2 (1.79)	4 (2.92)		
4	5 (2.01)	4 (3.57)	1 (0.73)		
Past medical history					
Hypertension, *n* (%)	111 (44.58)	55 (49.11)	56 (40.88)	χ^2^ = 1.690	0.194
Dyslipidemia, *n* (%)	28 (11.25)	14 (12.50)	14 (10.22)	χ^2^ = 0.321	0.571
Diabetes, *n* (%)	42 (16.87)	19 (16.96)	23 (16.79)	χ^2^ = 0.001	0.971
Stroke, *n* (%)	12 (4.82)	3 (2.68)	9 (6.57)	χ^2^ = 2.034	0.154
Medication history					
Antihypertension, *n* (%)	100 (40.16)	49 (43.75)	51 (37.23)	χ^2^ = 1.091	0.296
Lipid-lowering, *n* (%)	18 (7.23)	10 (8.93)	8 (5.84)	-	0.639
Atorvastatin	6 (2.41)	3 (2.68)	3 (2.19)		
Rosuvastatin	12 (4.82)	7 (6.25)	5 (3.65)		
Antidiabetes, *n* (%)	42 (16.87)	18 (16.07)	24 (17.52)	χ^2^ = 0.092	0.762
Laboratory parameters					
HGB, g/L	154.00 (140.00, 164.00)	152.00 (140.75, 161.25)	155.00 (140.00, 165.00)	Z = −1.340	0.180
PLT, ×10^9^/L	201.00 (162.00, 241.00)	201.50 (164.00, 233.25)	197.00 (160.00, 252.00)	Z = −0.217	0.828
HCY, μmol/L	17.20 (13.75, 27.23)	16.60 (13.50, 22.83)	18.45 (14.38, 30.63)	Z = −1.607	0.108
TnI, μg/L	2.74 (0.38, 16.00)	2.23 (0.44, 8.00)	4.32 (0.36, 23.00)	Z = −1.376	0.169
CK-MB, ng/mL	35.57 (11.00, 112.00)	28.33 (8.64, 79.22)	45.99 (12.00, 182.00)	Z = −1.830	0.067
Myo, ng/mL	190.50 (59.00, 616.00)	138.90 (50.38, 468.50)	253.00 (69.00, 802.00)	Z = −2.229	0.026
NT-proBNP, pg/mL	180.00 (74.00, 674.00)	171.00 (67.75, 757.00)	191.00 (79.00, 546.00)	Z = −0.049	0.961
CRP, mg/L	3.87 (1.25, 10.15)	3.71 (1.09, 8.87)	3.87 (1.26, 12.10)	Z = −0.437	0.662
FDP, mg/L	1.50 (0.87, 3.79)	1.32 (0.89, 3.07)	1.58 (0.84, 4.36)	Z = −0.770	0.441
D-D, μg/mL	0.43 (0.24, 1.29)	0.40 (0.23, 1.13)	0.49 (0.24, 1.33)	Z = −0.864	0.388
FIB, g/L	2.71 (2.34, 3.10)	2.73 (2.38, 3.11)	2.66 (2.27, 3.10)	Z = −0.966	0.334
HbA1c, %	5.70 (5.30, 6.30)	5.70 (5.30, 6.37)	5.70 (5.30, 6.10)	Z = −0.847	0.397
Crea, μmol/L	68.00 (58.90, 79.30)	67.60 (59.40, 79.88)	69.80 (58.20, 79.00)	Z = −0.155	0.877
UA, μmol/L	354.00 (288.00, 428.00)	354.50 (288.00, 441.25)	352.00 (292.00, 418.00)	Z = −0.500	0.617
Glu, mmol/L	6.29 (5.13, 8.00)	6.30 (4.99, 7.76)	6.27 (5.25, 8.41)	Z = −0.734	0.463
TC, mmol/L	4.39 (3.58, 5.10)	4.26 (3.38, 5.00)	4.47 (3.70, 5.18)	Z = −1.774	0.076
TG, mmol/L	1.45 (0.95, 2.30)	1.38 (0.88, 2.14)	1.46 (1.01, 2.54)	Z = −1.191	0.234
HDL-C, mmol/L	1.02 (0.91, 1.19)	1.03 (0.89, 1.14)	1.01 (0.92, 1.21)	Z = −0.024	0.981
LDL-C, mmol/L	2.90 (2.25, 3.38)	2.79 (2.11, 3.28)	2.96 (2.40, 3.51)	Z = −1.984	0.047
Lp(a), mg/dL	16.64 (6.32, 38.17)	15.84 (5.14, 38.17)	16.89 (7.13, 37.98)	Z = −0.967	0.334
Non-HDL-C, mmol/L	3.16 (2.58, 3.86)	2.95 (2.41, 3.49)	3.40 (2.71, 4.06)	Z = −3.379	<0.001
ApoA1, g/L	1.19 (1.03, 1.36)	1.22 (1.04, 1.37)	1.16 (1.03, 1.33)	Z = −1.052	0.293
ApoB, g/L	0.91 (0.74, 1.06)	0.86 (0.65, 1.02)	0.94 (0.78, 1.10)	Z = −2.997	0.003
ApoB/A1	0.76 (0.60, 0.93)	0.68 (0.54, 0.85)	0.80 (0.65, 0.95)	Z = −3.552	<0.001
RLP-C, mmol/L	0.46 (0.31, 0.67)	0.44 (0.27, 0.54)	0.51 (0.32, 0.77)	Z = −3.009	0.003
NHHR	3.07 (2.41, 3.74)	2.84 (2.22, 3.58)	3.25 (2.62, 3.88)	Z = −3.119	0.002
LCI	17.30 (8.20, 32.80)	17.05 (6.88, 28.35)	17.70 (9.40, 34.90)	Z = −1.674	0.094
TC/HDL	4.15 (3.38, 4.86)	3.89 (3.21, 4.81)	4.25 (3.62, 4.88)	Z = −1.971	0.049
HDL/ApoA1	0.89 (0.82, 0.98)	0.89 (0.80, 0.99)	0.88 (0.83, 0.97)	Z = −0.478	0.633
LDH, U/L	379.00 (231.00, 502.00)	257.50 (198.75, 399.25)	432.00 (335.00, 554.00)	Z = −6.134	<0.001
LVEF, %	54.00 (48.00, 58.00)	55.00 (50.00, 58.00)	53.00 (48.00, 58.00)	Z = −1.771	0.077

BMI, body mass index; SBP, systolic blood pressure; DBP, diastolic blood pressure; CAD, coronary artery disease; HGB, hemoglobin; PLT, platelet; HCY, homocysteine; TnI, troponin I; CK-MB, creatine kinase-MB; MyO, myoglobin; NT-proBNP, n-terminal pro-B-type natriuretic peptide; CRP, C-reactive protein; FDP, fibrin degradation products; D-D, d-dimer; FIB, fibrinogen; HbA1c, hemoglobinA1c; Crea, creatinine; UA, uric acid; Glu, glucose; TC, total cholesterol; TG, triglycerides; HDL-C, high-density lipoprotein cholesterol; LDL-C, low-density lipoprotein cholesterol; Lp(a), lipoprotein (a); non-HDL-C, non-high-density lipoprotein cholesterol; ApoA1, apolipoprotein A1; ApoB, apolipoprotein B; ApoB/A1, apolipoprotein B/A1 ratio; RLP-C, remnant lipoprotein cholesterol; non-HDL-C/HDL-C ratio, NHHR; LCI, lipoprotein combine index; TC/HDL, TC to HDL-C ratio; HDL/ApoA1, HDL-C to ApoA1 ratio; LDH, lactate dehydrogenase; LVEF, left ventricular ejection fraction, Fisher's exact test.

### Coronary angiographic and OCT characteristics

There were no significant differences between the two groups in the number of diseased vessels, culprit vessels, lesion segments, or lesion length (all *P* > 0.05). However, the proportions of plaque rupture (76.64% vs. 27.68%), plaque erosion (21.90% vs. 8.04%), and thrombus formation (36.50% vs. 24.11%) were significantly higher in the high-risk group (*P* < 0.05). OCT quantitative analysis revealed that the high-risk group had thinner fibrous caps (0.15 vs. 0.22 mm), a smaller minimal lumen area (2.01 vs. 3.24 mm^2^), and a heavier lipid burden (270 vs. 90°) (all *P* < 0.05). Meanwhile, macrophage infiltration (34.31% vs. 18.75%, *P* = 0.006) and cholesterol crystals (68.61% vs. 25.00%, *P* < 0.001) were more common in the high-risk group, and both the incidence (72.99% vs. 55.36%, *P* = 0.004) and thickness (0.40 vs. 0.30 mm, *P* = 0.007) of calcification were significantly increased (Table 2).

**Table 2:** Coronary angiographic and OCT findings in Non-high-risk and high-risk plaque groups.

Variables	All *N* = 249	Non-high-risk *N* = 112	High-risk *N* = 137	Statistic	*P*-value
Coronary angiography					
Number of diseased vessels, *n* (%)					
Single-vessel	164 (65.86)	75 (66.96)	89 (64.96)	χ^2^ = 0.110	0.740
Double-vessel	66 (26.51)	26 (23.21)	40 (29.20)	χ^2^ = 1.132	0.287
Triple-vessel	19 (7.63)	11 (9.82)	8 (5.84)	χ^2^ = 1.386	0.239
Culprit vessel, *n* (%)				χ^2^ = 1.988	0.370
LAD	119 (47.79)	59 (52.68)	60 (43.80)		
LCX	38 (15.26)	16 (14.29)	22 (16.06)		
RCA	92 (36.95)	37 (33.04)	55 (40.15)		
Lesion segment, *n* (%)				χ^2^ = 2.089	0.352
Proximal	170 (68.27)	79 (70.54)	91 (66.42)		
Mid	67 (26.91)	30 (26.79)	37 (27.01)		
Distal	12 (4.82)	3 (2.68)	9 (6.57)		
OCT findings					
Plaque rupture	136 (54.62)	31 (27.68)	105 (76.64)	χ^2^ = 59.603	<0.001
Plaque erosion	39 (15.66)	9 (8.04)	30 (21.90)	χ^2^ = 8.964	0.003
Thrombus, *n* (%)	77 (30.92)	27 (24.11)	50 (36.50)	χ^2^ = 4.428	0.035
Lesion length, mm	24.40 (21.30, 29.80)	25.000 (21.58, 31.40)	23.90 (21.00, 29.80)	Z = −0.890	0.374
FCT, mm	0.18 (0.12, 0.25)	0.22 (0.13, 0.36)	0.15 (0.12, 0.20)	Z = −4.449	<0.001
TCFA, *n* (%)	10 (4.02)	7 (6.25)	3 (2.19)	χ^2^ = 1.687	0.194
Lipid arc ( °)	180.00 (90.00, 270.00)	90.00 (70.00, 180.00)	270.00 (180.00, 360.00)	Z = −9.052	<0.001
LA ≥ 90°	196 (78.72)	59 (52.68)	137 (100.00)	χ^2^ = 82.361	<0.001
LA > 180°	104 (41.77)	18 (16.07)	86 (62.77)	χ^2^ = 55.260	<0.001
Macrophage, *n* (%)	68 (27.31)	21 (18.75)	47 (34.31)	χ^2^ = 7.512	0.006
Cholesterol crystal, *n* (%)	122 (49.00)	28 (25.00)	94 (68.61)	χ^2^ = 46.904	<0.001
Microvessels, *n* (%)	41 (16.47)	21 (18.75)	20 (14.60)	χ^2^ = 0.772	0.380
MLA, mm^2^	2.62 (1.44, 4.28)	3.24 (1.80, 5.15)	2.01 (1.33, 3.45)	Z = −3.850	<0.001
MLA < 3.5 mm^2^, *n* (%)	164 (65.86)	60 (53.57)	104 (75.91)	χ^2^ = 13.680	<0.001
Reference lumen area	7.12 (5.05, 9.33)	6.91 (4.66, 8.86)	7.54 (5.32, 9.65)	Z = −1.975	0.048
Fibrous plaque, *n* (%)	246 (98.80)	111 (99.11)	135 (98.54)	χ^2^ = 0.000	1.000
Calcification, *n* (%)	162 (65.06)	62 (55.36)	100 (72.99)	χ^2^ = 8.431	0.004
Thickness, mm	0.40 (0.00, 0.50)	0.30 (0.00, 0.50)	0.40 (0.20, 0.50)	Z = −2.708	0.007
Length, mm	4.00 (0.00, 5.00)	4.00 (0.00, 6.00)	4.00 (3.00, 5.00)	Z = −1.004	0.315
High plaque, *n* (%)	137 (55.02)	0 (0.00)	137 (100.00)	χ^2^ = 249.000	<0.001

LAD, left anterior descending artery; LCX, left circumflex artery; RCA, right coronary artery; FCT, fibrous cap thickness; TCFA, thin-cap fibroatheroma; LA, lipid arc; MLA, minimal lumen area; RLA, reference lumen area.

### Independent associations of ApoB/A1 and non-HDL-C with high-risk plaque formation

**Figure 3:**
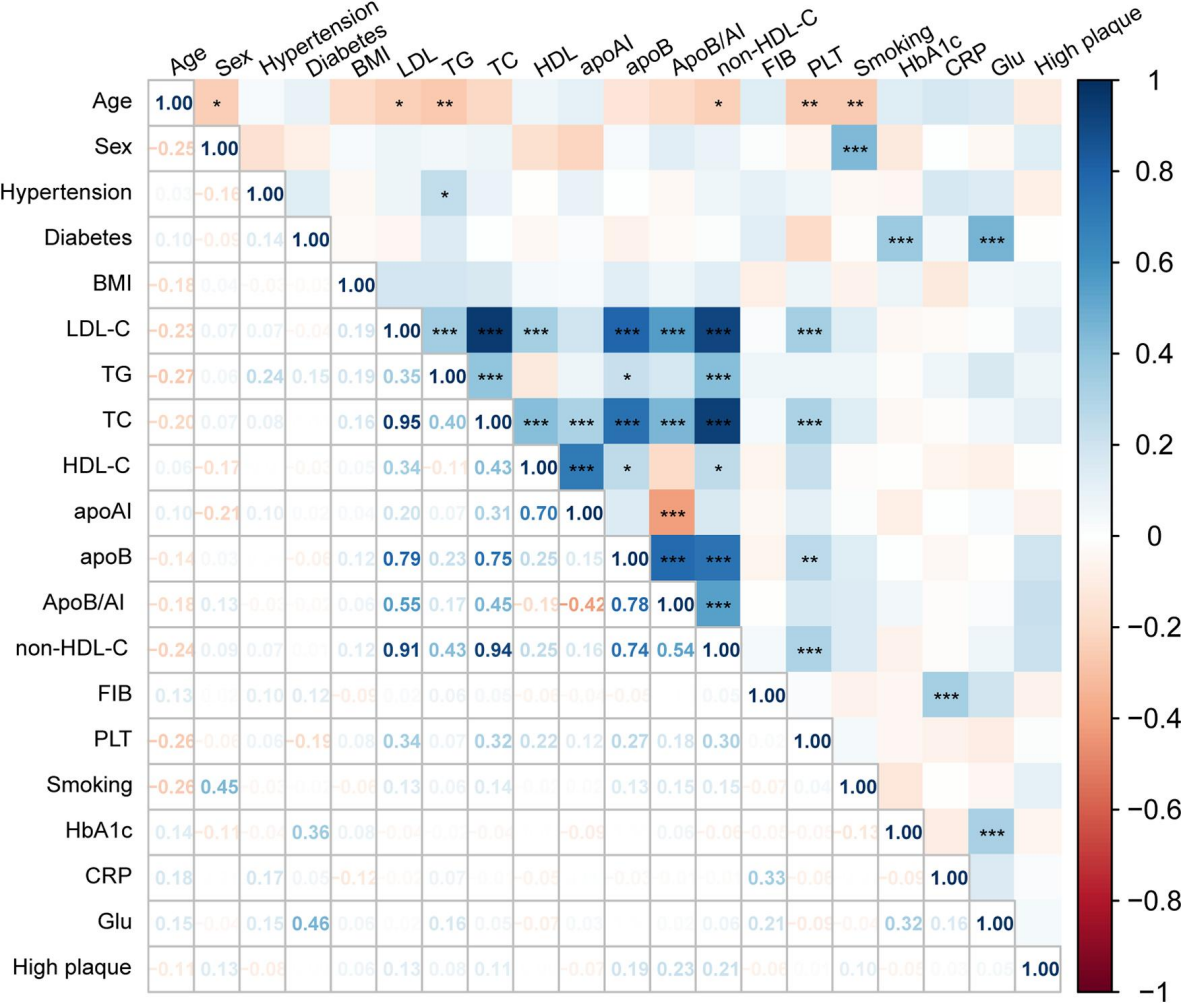
Spearman correlation between lipid parameters and high-risk plaque formation. Heatmap illustrating the correlations between lipid parameters and OCT-characterized high-risk plaques. Red indicates positive and blue negative correlations.

**Figure 4:**
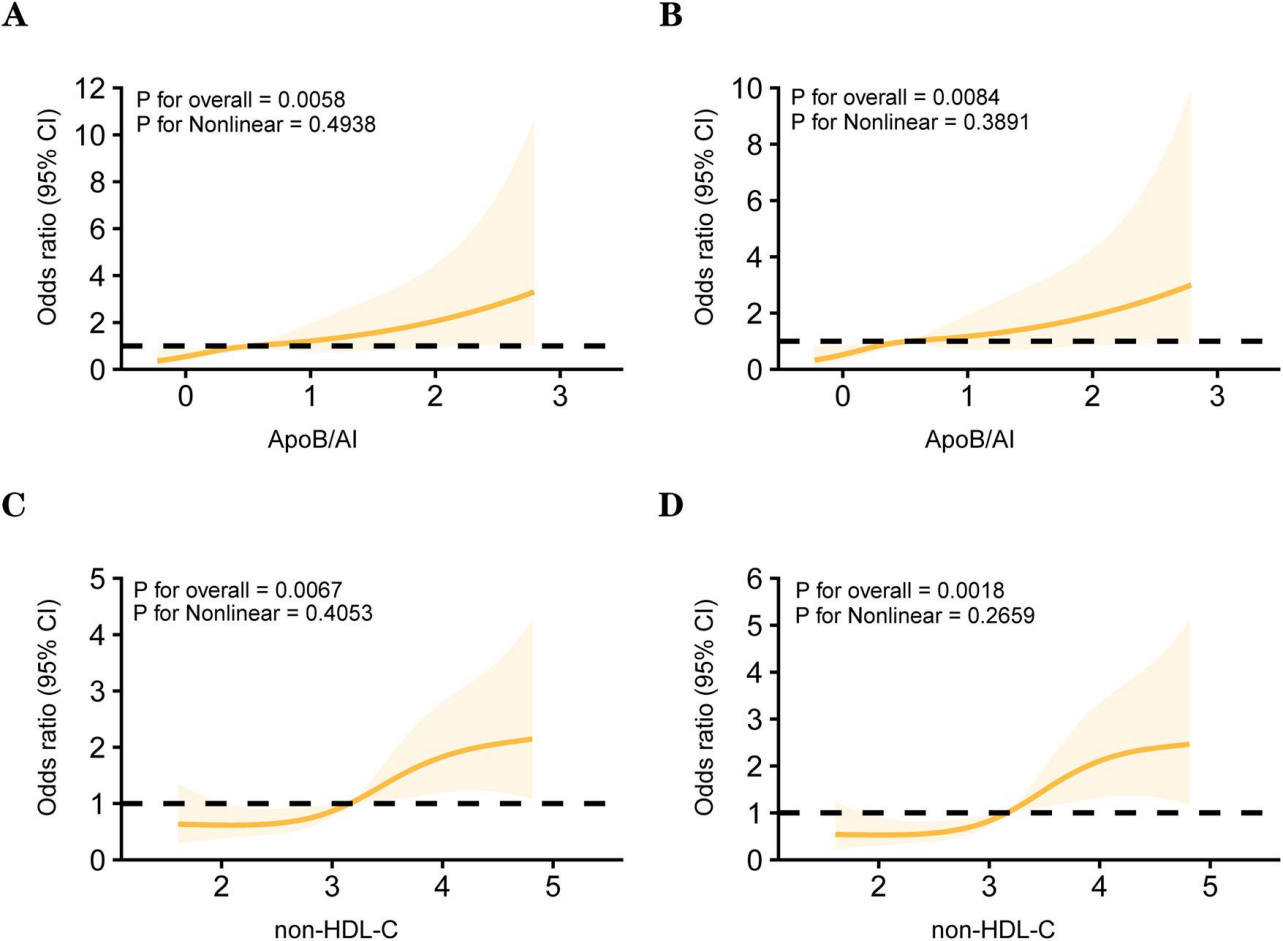
Restricted cubic spline (RCS) models between lipid parameters and the high-risk plaque. Panels **(A)** and **(B)** depict the associations between ApoB/A1 and high-risk plaque in unadjusted and multivariable-adjusted models, respectively. Panels **(C)** and **(D)** show the corresponding associations for non-HDL-C.

Spearman correlation analysis further demonstrated consistent trends among lipid parameters (Figure 3). Non-HDL-C, ApoB, and ApoB/A1 were strongly correlated with LDL-C and TC (r = 0.70–0.95, *P* < 0.001) and inversely correlated with HDL-C and ApoA1 (r ≈ −0.4, *P* < 0.01). Both non-HDL-C and ApoB/A1 showed a modest but significant positive correlation with high-risk plaques (r ≈ 0.20, *P* < 0.05). After excluding variables with severe multicollinearity, both univariate and multivariate logistic regression analyses identified non-HDL-C (OR = 3.023, 95% CI: 1.238–7.378, *P* = 0.015) and ApoB/A1 (OR = 3.688, 95% CI: 1.211–11.230, *P* = 0.022) as independent risk factors for high-risk plaque formation (Table 3). As shown in Table 4, in the unadjusted model, ApoB/A1 (OR = 4.038, 95% CI: 1.728–9.436) and non-HDL-C (OR = 1.558, 95% CI: 1.185–2.048) were significantly associated with the presence of high-risk plaques (all *P* < 0.05). These associations remained robust after progressive adjustment for demographic and clinical covariates (Model 3: ApoB/A1 OR = 3.726, 95% CI: 1.534–9.051, *P* = 0.004; non-HDL-C OR = 1.623, 95% CI: 1.192–2.211, *P* = 0.002). Tertile-based stratified analyses further demonstrated a progressive increase in high-risk plaque risk across higher levels of both indices (*P* for trend <0.01). RCS analysis revealed a positive linear relationship between ApoB/A1 (Panels A and B) and non-HDL-C (Panels C and D) levels and the risk of high-risk plaque formation. The overall associations were statistically significant (*P* < 0.01), with no evidence of nonlinearity (*P* > 0.05) (Figure 4).

**Table 3:** Logistic regression analyses of factors associated with high-risk plaque.

Variables	Univariate analysis					Multivariate analysis				
	*β*	SE	Z	OR (95%CI)	*P*	β	SE	Z	OR (95%CI)	*P*
Sex	0.764	0.369	2.072	2.148 (1.042–4.426)	0.038	0.644	0.401	1.605	1.905 (0.867–4.182)	0.108
Age	−0.016	0.011	−1.446	0.984 (0.964–1.006)	0.148					
BMI	0.007	0.040	0.171	1.007 (0.931–1.089)	0.864					
Smoke	0.425	0.258	1.647	1.529 (0.923–2.535)	0.099					
STEMI	0.921	0.313	2.944	2.511 (1.360–4.635)	0.003	0.654	0.352	1.858	1.923 (0.965–3.833)	0.063
Hypertension	−0.333	0.257	−1.298	0.716 (0.433–1.185)	0.194					
Dyslipidemia	−0.227	0.401	−0.566	0.797 (0.363–1.750)	0.571					
Diabetes	−0.013	0.340	−0.037	0.988 (0.507–1.923)	0.971					
HCY	0.003	0.006	0.403	1.003 (0.990–1.015)	0.687					
Antihypertension	−0.271	0.260	−1.044	0.762 (0.458–1.269)	0.297					
Antidiabetes	0.104	0.342	0.303	1.109 (0.568–2.167)	0.762					
TnI	0.022	0.011	1.884	1.022 (0.999–1.045)	0.060					
CKMB	0.002	0.001	1.910	1.002 (1.000–1.004)	0.056					
Myo	0.001	0.000	2.212	1.001 (1.001–1.002)	0.027	0.001	0.000	1.663	1.001 (1.000–1.002)	0.096
NT-proBNP	−0.000	0.000	−1.865	1.000 (1.000–1.000)	0.062					
CRP	0.006	0.006	0.912	1.006 (0.994–1.018)	0.362					
FDP	0.003	0.011	0.323	1.003 (0.982–1.025)	0.747					
D-D	−0.003	0.023	−0.112	0.997 (0.954–1.043)	0.911					
FIB	−0.151	0.171	−0.881	0.860 (0.615–1.203)	0.378					
Crea	0.001	0.005	0.206	1.001 (0.991–1.011)	0.837					
UA	−0.001	0.001	−0.531	0.999 (0.997–1.002)	0.595					
Glu	0.010	0.037	0.279	1.010 (0.939–1.087)	0.780					
TG	0.114	0.086	1.328	1.121 (0.947–1.326)	0.184					
Lp(a)	0.003	0.005	0.716	1.003 (0.994–1.013)	0.474					
non-HDL-C	0.444	0.139	3.180	1.558 (1.185–2.048)	0.001	1.106	0.455	2.429	3.023 (1.238–7.378)	0.015
ApoB/A1	1.396	0.433	3.223	4.038 (1.728–9.436)	0.001	1.305	0.568	2.297	3.688 (1.211–11.230)	0.022
NHHR	0.426	0.134	3.183	1.532 (1.178–1.991)	0.001					
RLP-C	1.055	0.373	2.830	2.872 (1.383–5.964)	0.005	0.670	0.477	1.403	1.953 (0.767–4.977)	0.161
LVEF	−0.021	0.019	−1.085	0.979 (0.943–1.017)	0.278					

**Table 4:** Associations of ApoB/A1 and non-HDL-C with high-risk plaque across different models.

Variables	Model 1		Model 2		Model 3	
	OR（95%CI）	*P*	OR（95%CI）	*P*	OR（95%CI）	*P*
ApoB/A1	4.038 (1.728–9.436)	<0.001	3.629 (1.525–8.636)	0.004	3.726 (1.534–9.051)	0.004
Tertile						
1	1.000（Reference）		1.000（Reference）		1.000（Reference）	
2	1.661 (0.901–3.062)	0.104	1.634 (0.873–3.057)	0.125	1.595 (0.840–3.031)	0.154
3	2.735 (1.449–5.163)	0.002	2.535 (1.322–4.860)	0.005	2.655 (1.358–5.188)	0.004
Non-HDL-C	1.558 (1.185–2.048)	0.001	1.535 (1.151–2.049)	0.004	1.623 (1.192–2.211)	0.002
Tertile						
1	1.000（Reference）		1.000（Reference）		1.000（Reference）	
2	0.935 (0.509–1.717)	0.828	0.934 (0.497–1.756)	0.832	0.986 (0.501–1.940)	0.968
3	2.828 (1.479–5.407)	0.002	2.838 (1.429–5.638)	0.003	3.254 (1.552–6.823)	0.002

Model 1: Unadjusted.

Model 2: adjust: sex, smoke, hypertension, diabetes, age, BMI.

Model 3: adjust: sex, smoke, hypertension, diabetes, age, BMI, lipid-lowing drugs, antihypertension, antidiabetes, crea, UA.

### Subgroup analysis of the association between ApoB/A1, non-HDL-C, and high-risk plaques

**Figure 5:**
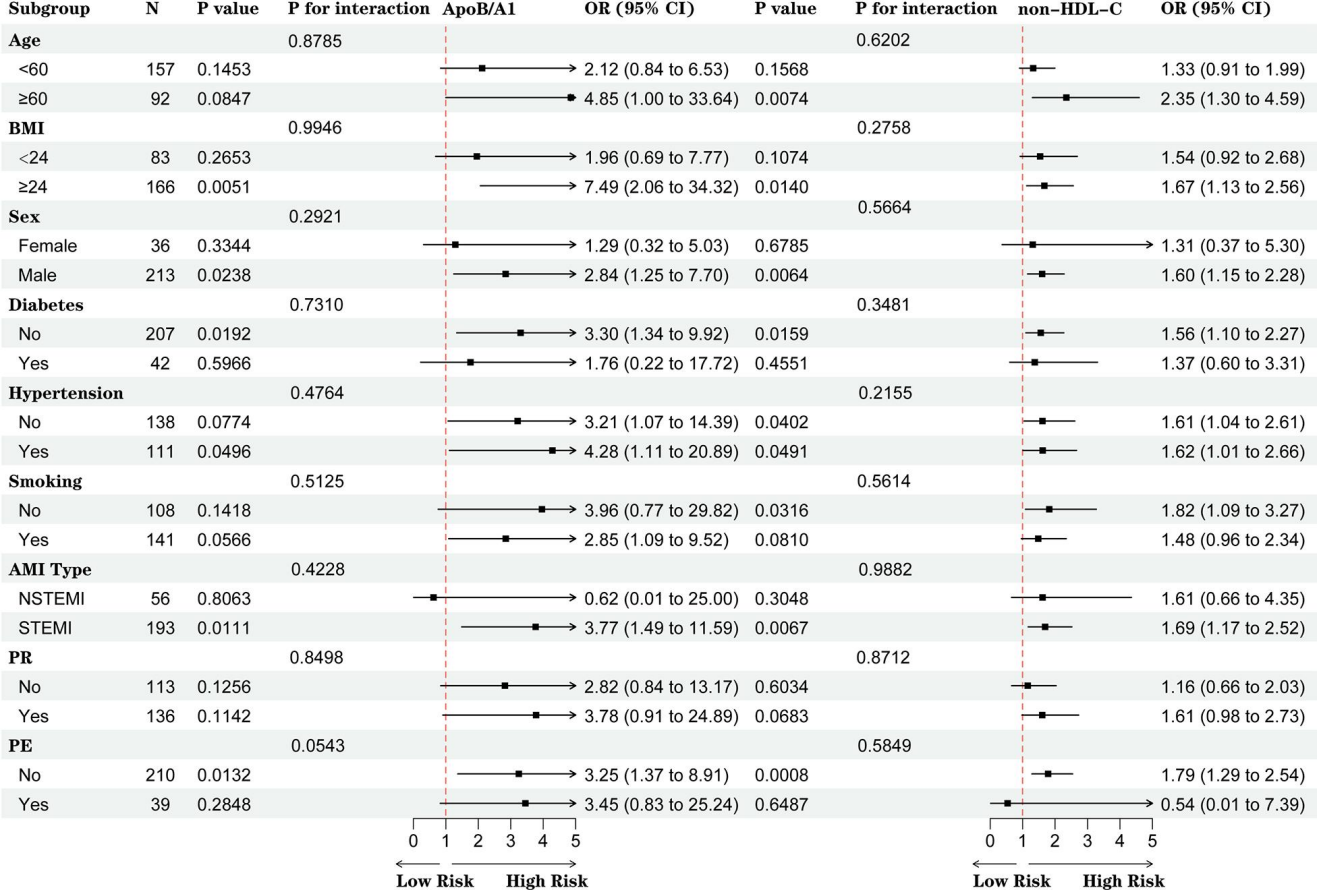
Subgroup analysis of the associations between ApoB/A1 and non-HDL-C levels and high-risk plaque formation.

No significant interactions were observed between ApoB/A1 or non-HDL-C levels and clinical subgroups (all *P* for interaction >0.05). However, elevated ApoB/A1 was more strongly associated with the presence of high-risk plaques among patients aged ≥60 years, with BMI ≥24 kg/m^2^, male sex, hypertension, and STEMI (all *P* < 0.05) (Figure 5).

### Incremental value of ApoB/A1 and non-HDL-C in identifying high-risk plaques

**Figure 6:**
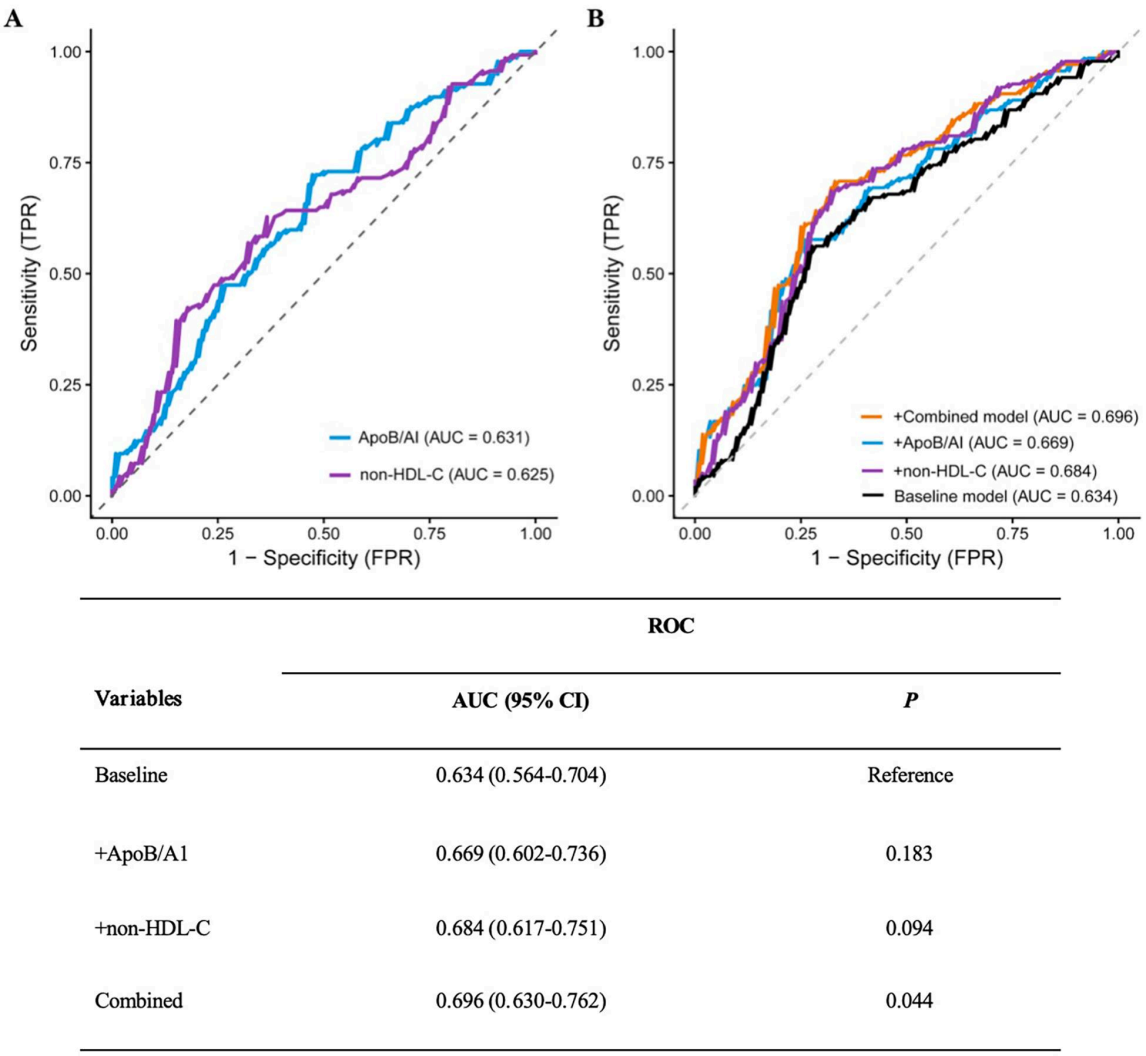
ROC curves and DeLong test of the baseline risk model with different added indicators. **(A)** Comparison of the ROC curves for ApoB/AI and non-HDL-C as individual indicators. **(B)** Comparison of the ROC curves for the baseline model versus models with the added inclusion of ApoB/AI, non-HDL-C, and their combination.

ROC curve analysis demonstrated that the AUC of ApoB/A1 was 0.631, slightly higher than that of non-HDL-C (0.625). Incorporating ApoB/A1 or non-–HDL-C individually, or simultaneously, into the baseline model improved its discriminative performance, with the combined model showing the greatest enhancement (AUC increased from 0.634 to 0.696, 95% CI: 0.630–0.762). The DeLong test confirmed that this improvement was statistically significant (*P* < 0.05), suggesting that the combination of ApoB/A1 and non-HDL-C contributes to better identification of high-risk plaques (Figure 6).

**Figure 7:**
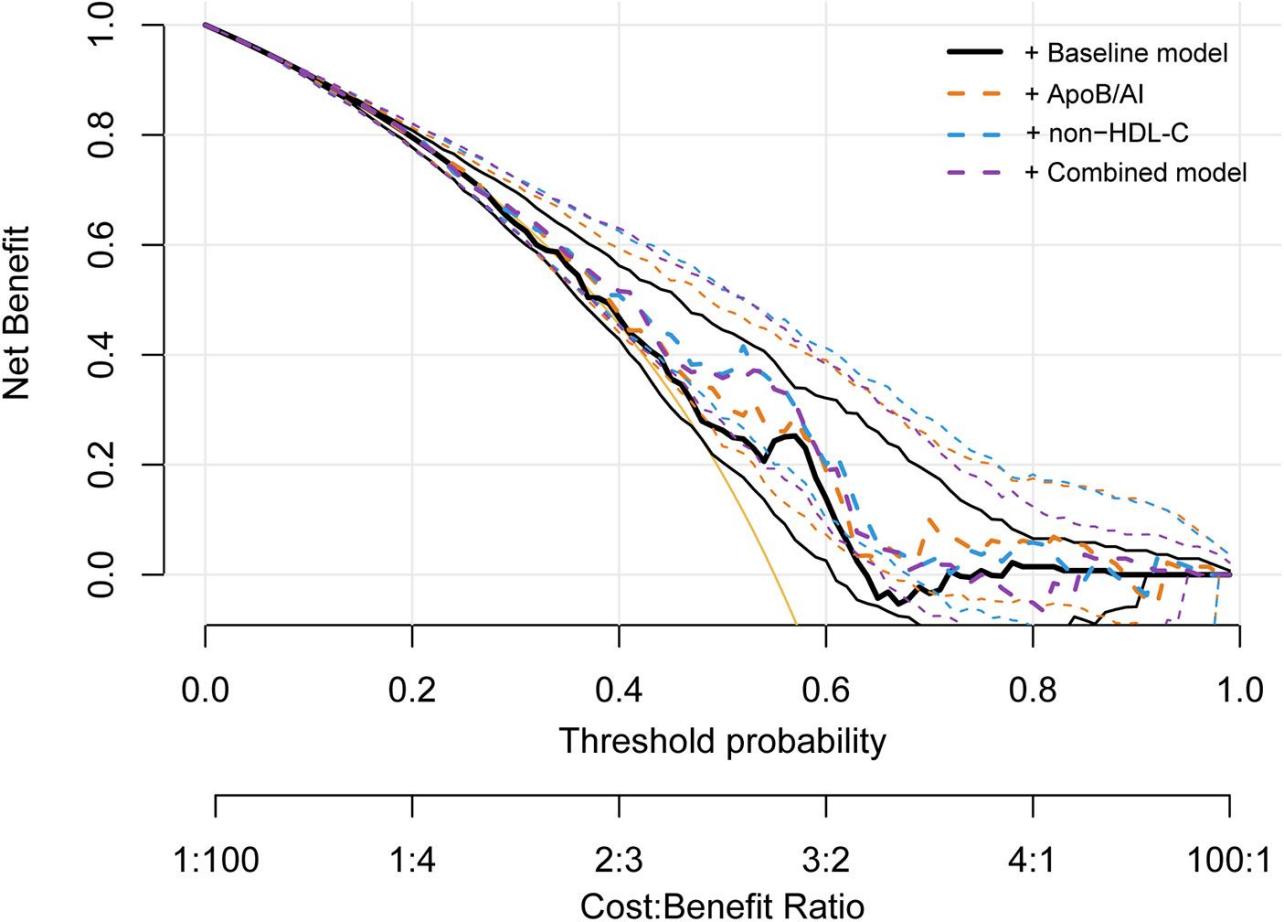
Decision curve analysis for evaluating the clinical utility of ApoB/A1 and non-HDL-C models in identifying high-risk plaques.

Further assessment using the IDI and NRI indices supported these findings. Adding ApoB/A1 or non-HDL-C alone significantly enhanced the discriminative ability of the baseline model (IDI: 0.042 and 0.051; NRI: 0.343 and 0.493, respectively; all *P* < 0.01). The improvement was most pronounced when both parameters were included simultaneously (IDI = 0.063, NRI = 0.515; both *P* < 0.001), indicating a synergistic effect in model optimization (Table 5). DCA further demonstrated that the baseline model provided limited clinical net benefit in distinguishing high-risk plaques. The inclusion of ApoB/A1 and/or non-HDL-C substantially increased net benefit across a wide range of threshold probabilities, with the combined model achieving the greatest clinical utility between thresholds of approximately 0.3 and 0.8 (Figure 7).

**Table 5:** Incremental effect of adding different indicators to the baseline model for identifying high-risk plaques.

Variables	IDI		NRI (continuous)	
	Estimated value (95% CI)	*P*	Estimated value (95% CI)	*P*
Baseline	Reference		Reference	
+ApoB/A1	0.042 (0.018–0.067)	<0.001	0.343 (0.101–0.586)	0.006
+Non-HDL-C	0.051 (0.024–0.078)	<0.001	0.493 (0.252–0.733)	<0.001
Combined	0.063 (0.033–0.093)	<0.001	0.515 (0.275–0.756)	<0.001

## Discussion

This retrospective study investigated the association between non-traditional lipid parameters and high-risk plaques characterized by OCT imaging in patients with AMI. Among the evaluated lipid markers, both the non-traditional ApoB/A1 ratio and the traditional lipid parameter non-HDL-C were independently associated with high-risk plaque morphology, maintaining robustness after multivariable adjustment. Both parameters exhibited a linear positive correlation with plaque risk, with consistent associations across different clinical subgroups. Although ApoB/A1 exhibited slightly higher discriminatory ability than non-HDL-C, the latter provided greater incremental improvement when added to the baseline risk model. Notably, combining ApoB/A1 with non-HDL-C yielded the most significant enhancement in identifying and discriminating high-risk plaques.

ApoB/A1 reflects the equilibrium between pro-atherogenic lipoproteins (ApoB-associated particles) and anti-atherogenic lipoproteins (HDL composed of ApoA1) and is considered a superior lipid risk indicator to LDL-C (17). Previous studies have indicated that ApoB/A1 is closely associated with multivessel coronary artery disease and recurrent myocardial infarction risk. Intravascular ultrasound (IVUS) studies revealed that ApoA1 and ApoB independently predict necrotic core volume in coronary artery disease patients (18). Deng et al. confirmed ApoB/A1 as an independent predictor of plaque rupture, erosion, and thrombosis in ASCVD patients (19), while Du et al. reported its significant correlation with tissue prolapse volume under OCT, suggesting a direct association between ApoB/A1 and plaque vulnerability (20). The present study further validates the clinical value of ApoB/A1 in identifying high-risk plaque morphology in AMI patients. Mechanistically, ApoA1 maintains cellular homeostasis by mediating cholesterol reverse transport and exerts anti-inflammatory, antioxidant, and anti-platelet aggregation effects (21, 22). Conversely, as a key structural protein of LDL, VLDL, and Lp(a), ApoB facilitates cholesterol entry into the arterial intima, inducing macrophage infiltration, foam cell formation, and inflammatory cytokine release (23–25). Consequently, elevated ApoB/A1 ratios indicate an imbalance in cholesterol uptake and clearance. This imbalance may enhance plaque inflammatory activity and fibrous cap instability through pathways such as activating NF-*κ*B signaling, inhibiting ABCA1/ABCG1-mediated efflux, and promoting M1-type polarization, thereby increasing plaque vulnerability risk.

The anti-atherosclerotic properties of HDL-C primarily stem from its pivotal role in reverse cholesterol transport (26), whereby it transports excess cholesterol from foam cells within arterial walls to the liver for metabolic clearance (27). Concurrently, it exerts biological effects, including antioxidant, anti-inflammatory, anti-apoptotic actions, and promotion of endothelial repair (28, 29). Voros et al. observed that small-particle HDL correlates with higher plaque burden and non-calcified plaque count, suggesting that HDL dysfunction may diminish its protective role in stabilizing plaques (30). Conversely, non-HDL-C represents the aggregate cholesterol burden of all atherogenic lipoproteins containing ApoB [including LDL, VLDL, IDL, and Lp(a)], and is now established by international guidelines as a key lipid target alongside LDL-C (31). Reddy et al. demonstrated via IVUS that elevated non-HDL-C levels positively correlate with necrotic core volume and overall plaque burden (32); Erlinge et al. further revealed a close association with the coronary artery lipid core burden index (33). The present study similarly indicates that non-HDL-C remains significantly correlated with high-risk plaques after adjusting for multiple confounding factors.

This study also found that although both ApoB/A1 and non-HDL-C were positively correlated with high-risk plaque formation, the effect of non-HDL-C remained stable across subgroups, whereas the risk association of ApoB/A1 was stronger in older individuals (≥60 years), overweight subjects (BMI ≥24 kg/m^2^), male patients, and those with STEMI. This pattern indicates that ApoB/A1 more sensitively reflects plaque instability in populations with greater metabolic burden and acute events (34, 35), whereas non-HDL-C, as a comprehensive indicator of overall atherosclerotic lipoprotein burden, is less susceptible to external confounding factors and thus provides a more stable reflection of high-risk plaque burden (36).

OCT morphological analysis further supports the aforementioned mechanistic hypothesis. Overall, high-risk plaques exhibited more pronounced inflammatory and lipid-rich characteristics. Notably, the high proportion of plaque erosion observed in the high-risk plaque group in this study appears to contrast with findings from Dai et al., who reported that plaque erosion in STEMI patients typically presented with lower lipid content, thicker fibrous caps, and a higher proportion of female patients (37). However, the high-risk plaque definition employed in this study (lipid arc ≥90°, thin cap, rupture/thrombosis ≥2 criteria) favors lesions with pronounced lipid and thrombus burden. Consequently, some eroded lesions with high lipid arc were also classified as high-risk. Furthermore, the study population predominantly comprised male patients (85.54%) and STEMI patients (77.51%), the latter typically exhibiting a more pronounced inflammatory and coagulation activation background (38, 39), rendering them more susceptible to erosive lesions with substantial lipid deposition. This demographic discrepancy may account for the phenotypic inconsistencies observed relative to prior studies. Concurrently, the low detection rate of TCFA (2.19%) in this study may relate to lesions predominantly having progressed to the terminal stages of rupture or thrombosis (40). Deng et al. confirmed that cholesterol crystals are prevalent in AMI culprit lesions and are closely associated with plaque vulnerability (41). Their sharp edges can directly cause mechanical damage to the fibrous cap, triggering rupture events. The significantly elevated proportion of cholesterol crystals in the high-risk group in this study supports this view. Shi et al. demonstrated that massive macrophage infiltration is associated with plaque rupture (42). This aligns with our findings of marked macrophage aggregation in high-risk plaques, suggesting local inflammatory activation as a key driver of plaque instability. Notably, calcification may coexist with either rupture or erosion, rather than being mutually exclusive (43). Early microcalcifications frequently originate from inflammatory deposits at the periphery of necrotic cores, leading to localized stress concentration and precipitating rupture (44). Late macrocalcifications, conversely, reflect a reparative response, stabilizing the plaque by reinforcing the fibrous cap structure (45, 46). Consequently, increased calcification thickness in high-risk plaques may reflect the complex, dynamic coexistence of inflammatory and reparative processes.

### Strengths and limitations

This study has several strengths. High-resolution OCT enabled detailed and reliable characterization of culprit lesion morphology in AMI. In addition, the lipid profile was obtained immediately after admission and before any lipid-lowering therapy, thereby minimizing treatment-related interference. Importantly, the study incorporated a comprehensive lipid panel, allowing a more thorough evaluation of lipid-related determinants of plaque vulnerability. However, several limitations warrant consideration. The retrospective, single-center design and limited sample size may restrict causal inference and generalizability. The predominance of male patients may reduce applicability to women. Moreover, OCT imaging evaluates only culprit lesions and may underestimate the overall coronary plaque burden. Future studies with larger sample sizes, multicenter participation, and longitudinal follow-up are warranted to further confirm these findings.

## Conclusion

In conclusion, both the non-traditional ApoB/A1 and the traditional lipid parameter non-HDL-C demonstrated independent and linear associations with OCT-characterized high-risk plaques in AMI. Their combined assessment provided a more integrated representation of lipid-related plaque vulnerability features. These findings enhance the understanding of lipid–plaque interactions and may contribute to clinical evaluation, pending validation in prospective studies.

## Data Availability

The raw data supporting the conclusions of this article will be made available by the authors without undue reservation.
